# The immediate post-operative period following lung transplantation: mapping of nursing interventions

**DOI:** 10.1590/0104-1169.3626.2480

**Published:** 2014

**Authors:** Rayssa Thompson Duarte, Graciele Fernanda da Costa Linch, Rita Catalina Aquino Caregnato

**Affiliations:** 1 RN, Intern in Nursing, Hospital São Lucas, Pontifícia Universidade Católica do Rio Grande do Sul, Porto Alegre, RS, Brazil; 2 PhD, Adjunct Professor, Universidade Federal de Ciências da Saúde de Porto Alegre, Porto Alegre, RS, Brazil

**Keywords:** Lung Transplantation, Nursing Care, Postoperative Care, Nursing Process

## Abstract

**OBJECTIVES:**

to investigate the principle nursing interventions/actions, prescribed in the immediate post-operative period for patients who receive lung transplantation, recorded in the medical records, and to map these using the Nursing Interventions Classification (NIC) taxonomy.

**METHOD:**

retrospective documental research using 183 medical records of patients who received lung transplantation (2007/2012). The data of the patients' profile were grouped in accordance with the variables investigated, and submitted to descriptive analysis. The nursing interventions prescribed were analyzed using the method of cross-mapping with the related interventions in the NIC. Medical records which did not contain nursing prescriptions were excluded.

**RESULTS:**

the majority of the patients were male, with medical diagnoses of pulmonary fibrosis, and underwent lung transplantation from a deceased donor. A total of 26 most frequently-cited interventions/actions were found. The majority (91.6%) were in the complex and basic physiological domains of the NIC. It was not possible to map two actions prescribed by the nurses.

**CONCLUSIONS:**

it was identified that the main prescriptions contained general care for the postoperative period of major surgery, rather than prescriptions individualized to the patient in the postoperative period following lung transplantation. Care measures related to pain were underestimated in the prescriptions. The mapping with the taxonomy can contribute to the elaboration of the care plan and to the use of computerized systems in this complex mode of therapy.

## Introduction

Lung transplantation is a fundamental therapeutic option for the treatment of serious non-neoplasic pneumopathies such as advanced Chronic Obstructive Pulmonary Disease (COPD), pulmonary fibrosis, cystic fibrosis, and pulmonary hypertension in the final stage and when the predicted life expectancy is below two years^(^ 1 ^)^.

The surveying of the Brazilian Transplant Registry (RBT, in Portuguese), between January and December 2013 indicated that only 80 lung transplants were undertaken in Brazil, representing a little over 1% of the total transplants of solid organs undertaken. However, the "low" representativity of the country is not reflected in Río Grande do Sul (RS), as in this state of Brazil, during the same period, 31 of the 80 lung transplants took place; 38.75% of the operations, falling behind only the state of São Paulo, where 46.25% of lung transplants were undertaken^(^ 2 ^)^.

The success of the process - from procuring organs through to the transplants - depends on the involvement and on the work of the multidisciplinary team, promoting the comprehensiveness of the care provided by the team to this patient in the perioperative period^(^ 3 ^)^. This model of multidisciplinary work makes the relationship of nursing with the process of organ donation and transplantation clear, evidencing the role of the nurse in both the care role and the role of transplant coordinator^(^ 4 ^)^.

It stands out that both patients who need lung transplants and those who have already received this organ are in a situation of vulnerability. In the pre-transplantation period, these are weakened because they present dyspnea and fatigue in activities which require minimum effort, compromising their activities of daily living, and sometimes leading them to be hospitalized while on the waiting list^(^ 5 ^)^. In the postoperative period following lung transplantation, the patients are recovering from an invasive and complex procedure which evidences the need for intensive care, besides the physiological complications themselves to which the procedure exposes them, such as: reperfusion edema, acute rejection, chronic rejection, infection by cytomegalovirus (CMV) and cryptogenic organising pneumonia, as well as dehiscence or bronchial anastomatic stenosis^(^ 6 ^)^.

In order to facilitate critical reflection on the care practiced by the nurses, to underscore the assistance which the same provide to the patients, and to contribute to effectiveness in the communication and documentation of the clinical practice, the use of scientific taxonomies such as the Nursing Interventions Classification (NIC) in search of excellence of practice in nursing^(^ 7 ^-^ 8 ^)^ has been strengthened. 

It is understood that nursing interventions cover "any treatment based in the judgment and the clinical knowledge undertaken by the nurse in order to improve the patient/client outcomes"^(^ 9 ^)^.

In order to research the actions undertaken and documented by the nurse, the use of cross-mapping has increasingly spread in the field of nursing, as it allows the data present in the nursing process as non-standardized diagnoses, results and interventions to be analyzed and compared with the scientific references and taxonomies indicated^(^ 10 ^)^.

The use of standardized language, and the close relationship of nursing with the recipient, find scientific support for interlinking them, with the objective of providing better assistance, principally in the most delicate period of this process, during the immediate postoperative period, when the patient is admitted to the Intensive Care Unit (ICU). 

Based on access to this information, emphasis is placed on the need to investigate the nursing care measures which are most relevant to the assistance provided to the patient in the immediate postoperative period following lung transplantation, mapping them in accordance with the NIC. Thus, the comparison of the care steps prescribed by the professionals with the taxonomy indicated by academics confers greater credibility on the nurse's work, facilitating the insertion of this language in the nursing process undertaken in their day-to-day^(^ 11 ^)^.

The present study aims to investigate the principal nursing interventions/actions prescribed in the immediate postoperative period for patients who received lung transplants, based on records made in the medical records, and to map these with the NIC taxonomy. 

As a result, the topic's relevancy is justified by the strong role of nursing in the donation and transplantation process, by the scarcity of articles which are directly related to the issue, by the lack of current works in the area, and by the particular importance which the state of Rio Grande do Sul has in the progression and statistics regarding lung transplantation in the Brazilian scenario.

## Method

This is transversal quantitative retrospective documental field research undertaken in a hospital which is a center of excellence in transplantation, located in Rio Grande do Sul.

The population was made up of 183 medical records of patients who underwent lung transplantation in the period 2007 - 2012. A total of 69 medical records was excluded, 23 of which corresponded to transplants undertaken in the months between September and December 2012, as the inclusion of a new electronic record system blocked access to the data, and 46 of which did not contain nursing prescriptions for the immediate postoperative period (IPP) following lung transplantation. As a result there was a final sample of 114 medical records, 62.29% of the population. 

Data collection was undertaken in the period between November 2012 and February 2013. Two electronic spreadsheets were used for data collection, one being used for identifying the profile of patients who received lung transplantation, investigating the variables: year of the operation, patient age, sex, preoperative medical diagnosis, type of transplant, presence of the nursing process, the need for retransplantation, and death. The other spreadsheet compiled all of the nursing actions/interventions available to be prescribed by the nurse, through the electronic record system. In this way, each time that one of the interventions was prescribed for a patient, it was indicated in the spreadsheet, in the line corresponding to the patient. 

The Statistical Package for the Social Sciences (SPSS), version 17.0 software was used for statistical analysis; the data were grouped in accordance with the variables investigated, and descriptive analyses were undertaken. The continuous variables were described by mean and standard deviation, and the categorical variables, by simple frequency and percentages. For the cross-mapping^(^ 10 ^)^, the interventions were identified, and evaluated in accordance with their similarity in accordance with each one of the domains stipulated by the NIC taxonomy. 

The study was approved by the institution's Research Ethics Committee under a decision substantiated with CAAE N. 05915812.0.0000.5335. The ethical questions were upheld with a signed commitment for the use of the data by the researchers. 

## Results

In relation to the profile of the patients who received lung transplantation between the years of 2007 and 2012, it was identified that the majority were male (60.63%), with a mean age of 49.28 (±15.29) years old, the youngest patient being nine years old, and the oldest, 73. The most prevalent medical diagnosis was pulmonary fibrosis (31.57%). This profile can be seen in Table 1. 

**Table 1:** - Profile of patients who received lung transplants, 2007 - 2012 (N=114). Porto Alegre, RS, Brazil, 2013

Variables		N(%)
Age*		49.28 (± 15.29)
Sex, male		70 (61.40)
Medical diagnosis		
	Pulmonary fibrosis	36 (31.57)
	Emphysema	33 (28.94)
	Advanced COPD	15 (13.16)
	Respiratory failure	12 (10.53)
	Pulmonary hypertension	10 (8.8)
	Others	8 (7.02)
Type of Transplant		
	Deceased donor	112 (98.24)
	Living donor	2 (1.75)
Retransplantation (yes)		6 (5.26)
Death		57 (50)

* Variable described as Mean and Standard Deviation.

The actions available to be prescribed by the nurse, through the electronic record system, totaled 69 items. Of the prescribed actions, those which had a representativity of at least 30% in the sample were mapped, resulting in 26 prescribed actions with greater significance in the care for this clientele (described in Table 2). 

**Table 2:** - The most prevalent nursing care measures for recipients of lung transplantation in the immediate postoperative period, in the intensive care unit (N=114). Porto Alegre, RS, Brazil, 2013

Nursing Prescriptions	N(%)
Give bed bath and change electrodes	112 (98.0)
Change and identify equipment, I.V lines and cannulas every 72 hours	109 (95.61)
Apply and note the appearance of the catheter dressing	107 (93.85)
Apply chest tube dressing	107 (93.85)
Observe and communicate regarding warming and blood perfusion of the extremities	107 (93.85)
Control vital signs	106 (92.98)
Observe ventilatory pattern	103 (90.35)
Undertake oral hygiene with oral antiseptic	99 (86.84)
Wash hands before and after dealing with the patient	98 (85.96)
Measure drainage from the chest drain and empty the drainage collection chamber	97 (85.08)
Maintain the bed head elevated at 30°	96 (84.21)
Control permeability of the administration route	96 (84.21)
Maintain care for the Swan-Ganz catheter	96 (84.21)
Maintain the chest drain with continuous aspiration	95 (83.33)
Observe and communicate reduction in urinary output	95 (83.33)
Observe signs of rejection or complications of the graft (temperature curve, pain, bleeding, abdominal distention)	94 (82.45)
Aspiration of Endotracheal Tube (ETT) with closed system, or aspirate Orotracheal Tube (OTT)	93 (81.57)
Apply medium-chain triglycerides on bony prominences	92 (80.70)
Maintain convoluted foam mattress pad	92 (80.70)
Control and note parameters of ventilator	90 (78.94)
Administer flush + observe permeability of the MAP	82 (71.92)
Change the braid on the tube	81 (70.17)
Check the cuff pressure	79 (69.29)
Change the bacterial filter on the ventilator	78 (68.42)
Change closed aspiration system of the orotracheal tube	66 (57.89)
Change the patient’s position	35 (30)

Among the 26 actions found by the study, 24 were mapped. Among these, six (25%) are allocated in the NIC *basic physiological domain*, belonging to three different levels: level F (*facilitation of self-care*), three actions (12.5%); level C (*control of immobility*) two actions (8.3%); level B (control of elimination) one action (4.16%). The other 16 actions prescribed (66.7%) are part of the NIC *complex physiological domain*, and are also allocated in three different levels: level K (*respiratory control*), eight actions (33.3%); level N (*control of tissue perfusion*), six actions (25%), and level L (*control of skin/wounds*), two actions (8.3%). 

The actions related to the complex and basic physiological domains represented the larger part of the interventions in the sample studied, however, in addition to these, a further domain had significance in the mapping of the nursing prescriptions: the NIC *safety domain*, *level V: control of risk *with two actions (8%). There were, however, no actions in the NIC domains: behavioral, family, health system, and community. 

It was not possible to undertake the cross-mapping of two prescribed actions: *observe signs of rejection or complications of the graft (temperature curve, pain, bleeding, abdominal distention) *and *changing closed aspiration system of the orotracheal tube, *as these do not correspond to any NIC intervention/action. 

**Figure 1:**
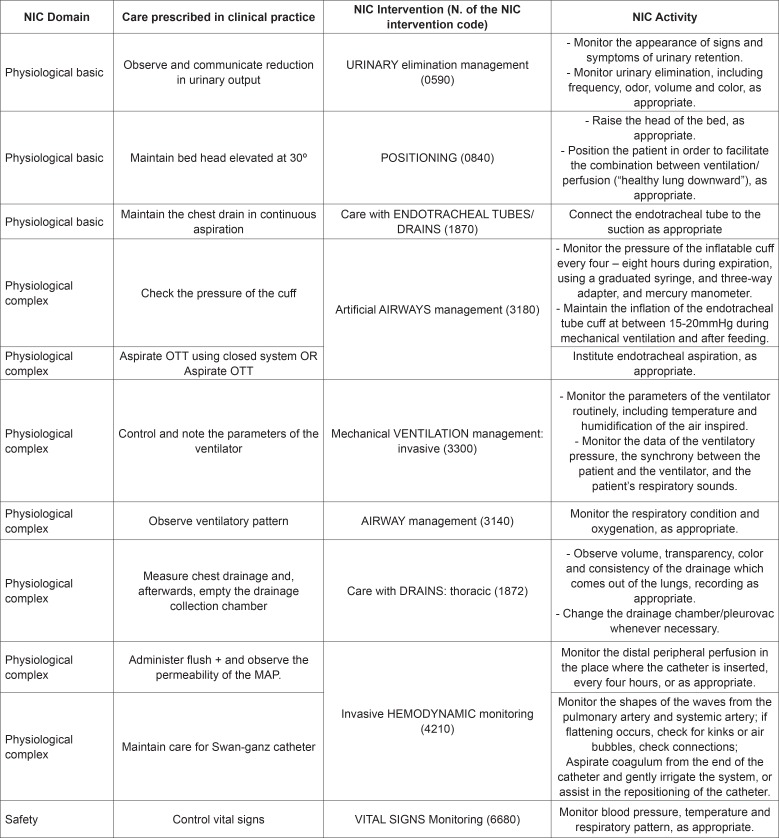
- NIC Domains, nursing actions prescribed for undertaking the care in the clinical practice in its original language and mapping with the corresponding NIC intervention and action

Of the actions mapped, Figure 1 presents only those related to the care prescribed by the nurses in the immediate postoperative period following lung transplantation. 

The interventions related to pain, such as: "evaluate pain" and "administer prescribed analgesics following the nurse's evaluation" were found in fewer than 16 (14%) prescriptions, representing, respectively, 7.89% and 6.14%. As they had a representativity below 30% in the sample, they were not mapped with the NIC actions.

## Discussion

The profile found in this study for the patients who received lung transplants showed a mean age among the patients of 50.19 (± 15.29) years old, and prevalence of the male sex (60.63%). The most prevalent base pathologies were: pulmonary fibrosis (35.63%), emphysema (20.62%) and COPD (4.3%), these being in accordance with other studies undertaken with the same target public^(^ 12 ^-^ 14 ^)^.

The nursing care prescribed for the persons who received lung transplants in the immediate postoperative period must follow a clinical reasoning based on the specific characteristics presented by the patients. For this, the nurse must recognize characteristics such as: the patient's primary diagnosis, preoperative condition, type of transplant undertaken, and the characteristics of the graft which impact on the care routines established^(^ 15 ^-^ 16 ^)^. The nurse's perception for early identification of signs and symptoms of postoperative complications directly influences the postsurgical prognosis^(^ 17 ^)^.

It is observed that the main interventions prescribed by the nurses directed towards respiratory care are indicated by the Center of Disease Control Bundle for prevention of ventilator-associated pneumonias (VAP)^(^ 18 ^)^. Care measures such as: undertaking oral hygiene with oral antiseptic, maintaining the bed head elevated at 30°, checking the pressure of the cuff and Endotracheal tube (ETT) aspiration with a closed system are in accordance with the Brazilian National Health Surveillance Agency's manual of respiratory tract infections and with a convergent-assistential qualitative study which presents, respectively, oral hygiene care with chlorhexidine 0.12%, elevation of the bed head between 30-45º, pressure of the cuff between 20-30 cm H_2_O; and care with aspiration of the secretions^(^ 19 ^)^. Such care measures for prevention of VAP are essential in order to avoid respiratory infections and, consequently, the acute rejection of the graft^(^ 15 ^,^ 20 ^-^ 21 ^)^.

It was identified that the main actions prescribed by the nurses were of general care for the postoperative period of any major surgery, such as: *bed bath, applying medium-chain triglycerides (MCT) on the bony prominences, maintain the convoluted foam mattress pad, *and *change the patient's position every two hours. *Although the above-mentioned interventions are important for the patients to progress well in the postoperative period, they may not be compatible with the postoperative period experienced by the recipient of a lung transplant, evidencing a lack of clinical reasoning and of the undertaking of automatized care.

The majority of actions prescribed for the postoperative period following lung transplantation are similar to the care provided to patients in the postoperative period of other major operations, such as: liver transplants, nephrectomies and cardiac surgery, which emphasize the management of pain and vital signs, early mobilization, respiratory management through the aspiration of secretions, monitoring of the respiratory pattern, positioning of the patient, and cardiovascular management undertaken through the maintenance of care with catheters, and surveillance of blood losses^(^ 22 ^-^ 23 ^)^.

In this study, the care measures mentioned above were mapped with the following interventions: *Monitoring of vital signs (6680); Artificial airway management (3180); Mechanical ventilation management: invasive (3300); Airway management (3140); Positioning (0840) *and *Invasive hemodynamic monitoring (4210).*

Nursing care in the postoperative period must be individualized, but can follow the same clinical reasoning, based on signs and symptoms, for providing safe care^(^ 24 ^)^. For this, hemodynamic monitoring, recognition of hypovolemia, control of the ventilatory strategy, aspiration of secretions and pain management must be taken into consideration.

The management of the pain was underestimated in many prescriptions, in spite of being frequently mentioned in many studies as the principal postoperative care measure following major surgery. Pain is considered the fifth vital sign to be evaluated, and lung transplantation is major surgery, with combined anesthesia and maintenance of the peridural catheter in the Immediate Postoperative Period (IPP)^(^ 15 ^,^ 22 ^,^ 24 ^)^. However, in the present study only 16 prescriptions were undertaken in relation to pain, representing 14% of the care measures prescribed. 

In spite of it being recommended that care with immunosuppression should start in the preoperative period, the need is noted for maintaining and improving these care measures in the postoperative period: in the same way that we must be alert for signs of acute rejection, reperfusion injuries, bleeding, arrhythmias, and signs of infection, especially in the first 48 hours^(^ 25 ^)^. 

However, based on the undertaking of the cross-mapping, it was possible to identify the main nursing interventions which are used in this clinical scenario. This being the case, this research can help the nurse in making clinical decisions for the prescription of specific care measures for recipients of lung transplantation. In the same way, the results may be used in other centers of lung transplantation. 

## Conclusion

This study made it possible to identify that the principal nursing interventions prescribed in the postoperative period following lung transplantation are similar to the interventions prescribed in major surgery, such as other modes of transplants and cardiac surgery. Only two of the interventions prescribed by the nurses could not be mapped, due to the lack of a similar care measure in the NIC; the others were mapped easily. Among the interventions mapped due to similarities, the majority are located in the complex and basic physiological domains of the NIC, evidencing that the care provided in the Intensive Care Unit (ICU) is directed to hemodynamic and respiratory support, placing emphasis on the prevention of VAP. However, pain management, essential care in the immediate postoperative period, was underestimated in the prescriptions. In the same way, specific care measures related to lung transplantation, in relation to acute rejection and immunosuppression, were also valued less than some general care measures.

It was identified that no prescription had interventions geared towards support for family members, different from what is stipulated by the Resolution of the Collegiate Directorate (RDC, in Portuguese) number 7, which is about the regulation of Intensive Care Units (ICUs) and indicates that patients' family members or companions must receive all the guidance and information necessary. Attention is also drawn to the fact that approximately 28% of the 160 medical records initially included in the study did not contain nursing prescriptions in the immediate postoperative period following lung transplantation. 

The identification of the most frequent nursing interventions in the care provided in the immediate postoperative period to the recipient of lung transplantation, and the later mapping of the same with the NIC taxonomy may contribute to the clinical practice of the nurses who work in this area. The cross-mapping can assist in the elaboration of care plans or protocols based in clinical reasoning, in the use of electronic nursing records, and also for qualifying and individualizing the care given to this clientele.
